# Systematic Exploration of the Synthetic Parameters for the Production of Dynamic VO_2_(M1)

**DOI:** 10.3390/molecules26154513

**Published:** 2021-07-27

**Authors:** Giulia Bragaggia, Andrea Cacciatore, Elisa Poffe, Claudia Capone, Federico Zorzi, Valerio Causin, Silvia Gross

**Affiliations:** 1 Dipartimento di Scienze Chimiche, Università degli Studi di Padova, Via Marzolo 1, 35131 Padova, Italy; giulia.bragaggia@unipd.it (G.B.); a.cacciatore@italcementi.it (A.C.); elisa.poffe@gmail.com (E.P.); valerio.causin@unipd.it (V.C.); 2 INSTM, Consorzio Interuniversitario per la Scienza e Tecnologia dei Materiali, Via Giusti 9, 50121 Firenze, Italy; 3 Italcementi S.p.A., HeidelbergCement Group, Via Stezzano 87, 24126 Bergamo, Italy; c.capone@italcementi.it; 4 CEASC, Centro di Analisi e Servizi per la Certificazione, Via Jappelli 1/A, 35131 Padova, Italy; federico.zorzi@unipd.it; 5 Dipartimento di Geoscienze, Università degli Studi di Padova, Via Gradenigo 6, 35131 Padova, Italy

**Keywords:** thermochromic materials, VO_2_(M1), VO_2_(R), metal–insulator transition (MIT), hydrothermal synthesis, functionalization

## Abstract

Thermochromic dynamic cool materials present a reversible change of their properties wherein by increasing the temperature, the reflectance, conductivity, and transmittance change due to a reversible crystalline phase transition. In particular, vanadium (IV) dioxide shows a reversible phase transition, accompanied by a change in optical properties, from monoclinic VO_2_(M1) to tetragonal VO_2_(R). In this paper, we report on a systematic exploration of the parameters for the synthesis of vanadium dioxide VO_2_(M1) via an easy, sustainable, reproducible, fast, scalable, and low-cost hydrothermal route without hazardous chemicals, followed by an annealing treatment. The metastable phase VO_2_(B), obtained via a hydrothermal route, was converted into the stable VO_2_(M1), which shows a metal–insulator transition (MIT) at 68 °C that is useful for different applications, from energy-efficient smart windows to dynamic concrete. Within this scenario, a further functionalization of the oxide nanostructures with tetraethyl orthosilicate (TEOS), characterized by an extreme alkaline environment, was carried out to ensure compatibility with the concrete matrix. Structural properties of the synthesized vanadium dioxides were investigated using temperature-dependent X-ray Diffraction analysis (XRD), while compositional and morphological properties were assessed using Scanning Electron Microscopy, Energy Dispersive X-ray Analysis (SEM-EDX), and Transmission Electron Microscopy (TEM). Differential Scanning Calorimetry (DSC) analysis was used to investigate the thermal behavior.

## 1. Introduction

Thermochromic materials represent a wide and extensive class of materials that falls within the field of the chromogenic device technology [1]. They are particularly used to vary the throughput of visible light for several applications, e.g., smart windows [2,3]. These materials show a metal–insulator transition (MIT) phenomenon associated with resistivity, transmittance, conductivity, and reflectance changes [4], and are mainly employed to reduce buildings’ energy consumption. Buildings account for around 30–40% of the world’s total energy intake due to the excessive use of lighting, air conditioning, and heating [5,6]. The atmospheric content of carbon dioxide has risen from 315 ppm at the end of the 1950s to 419 ppm in 2021, with a range of uncertainty of 0.1 ppm [7], mostly due to the burning of fossil fuels and with relevant consequences for life on Earth [8]. Because of their high energy uptake, buildings represent one of the most crucial issues in this area and a large number of technologies has been explored, with particular focus on smart windows, which have variable throughput of solar energy and visible light that can lower energy consumption. Windows are known to be the most inefficient elements of buildings. Smart windows employ chromogenic materials [9], specifically those with electrochromic or thermochromic properties. A wide range of materials has been studied for this application, including titanium oxides (Ti_2_O_3_, Ti_3_O_5_) [10], perovskites (LnNiO_3_, Ln = Pr, Nd, Sm, Eu) [11], and vanadium oxides (V_2_O_3_, V_6_O_13_, V_4_O_7_, VO_2_) [12]. Within this context, vanadium dioxide, VO_2_, represents one of the most promising and widely studied inorganic thermochromic materials [13], since its polymorph VO_2_(M) shows a MIT at a temperature of *τ_c_* ≈ 68 °C [14]. This transition is followed by a reversible crystal structure transformation from a low-temperature insulator phase, VO_2_(M), to a high-temperature metal phase, VO_2_(R). As a consequence, a change in the optical properties of vanadium dioxide occurs, namely an increase in the reflectance and a decrease in the transmittance, in addition to a higher conductivity. Vanadium dioxide exists in 10 different polymorphs, among which the most common are VO_2_(B), VO_2_(M), and VO_2_(D), belonging to the monoclinic crystal system; VO_2_(A) and VO_2_(R), with tetragonal structures; and VO_2_(P), which belongs to the orthorhombic crystal system. The VO_2_(B) polymorph, space group C2/m, is an interesting cathode material for Li-ion batteries and shows a reversible structure switch from amorphous to crystalline phase under high pressure [15,16]; VO_2_(D), space group P2/c, is a new VO_2_ phase observed by Qu et al. [17] that can transform to VO_2_(R) at about 593 K [18]. 

The VO_2_(A) polymorph, space group P4_2_/ncm, is an intermediate phase between VO_2_(B) and VO_2_(R) and undergoes a MIT at 162 °C [19], while VO_2_(P), space group Pbnm, is a paramontroseite VO_2_ mineral that can be transformed to VO_2_(M) by fast annealing [20]. The VO_2_(M) and VO_2_(R) oxides, with space groups P2_1_/c and P4_2_/mnm, respectively, represent the most interesting polymorphs of vanadium dioxide, since they display a reversible phase transition near room temperature. Changes in the crystal structure due to both Peierls and Mott’ mechanisms [21] contribute to the phase transition. Furthermore, using micro-Raman spectroscopy, Shao et al. [22] revealed the presence of two monoclinic phases, VO_2_(M1) and VO_2_(M2), and a triclinic phase, VO_2_(T), induced by temperature or strain, that can promote the transition to VO_2_(R).

With the purpose of obtaining a pure VO_2_(M1) phase, hydrothermal synthesis was employed and optimization was carried out of several synthetic parameters, including the vanadium precursor/reducing agent molar ratio, the reaction time, and the temperature required for the formation of the phase-pure VO_2_(M1). Furthermore, the influences of different vanadium precursors and reducing agents were investigated. In view of a possible application as additive for concrete, functionalization with a silica layer was performed, with the purpose of enhancing the compatibility with concrete. A stability test at pH = 11 on the functionalized vanadium dioxide was implemented to mimic the alkaline concrete’s pH. Once optimal conditions were established, a scale-up process was implemented by maintaining the vanadium precursor/reducing agent molar ratio constant and increasing the molar ratio with respect to the solvent. These variations did not produce any difference in the XRD analyses.

Therefore, this synthesis proved to be fast, easy, reproducible, scalable, and low cost with respect to the synthetic routes reported in the literature. These syntheses involve the use of hazardous chemicals and/or long reaction times, two conditions not compliant with future industrial production.

This work was performed in collaboration with Italcementi S.p.A. within the “COOL IT, progetto finanziato a valere sul Fondo di Ricerca di Sistema Elettrico” project. Among the activities performed as part of this project, this research aimed at lowering buildings’ energy uptake by integrating inorganic VO_2_ powder into concrete.

## 2. Results and Discussion

### 2.1. Synthesis and Characterization of VO_2_(B) and Its Conversion to VO_2_(M1)

The samples were prepared via hydrothermal synthesis, since this route is well-established [23,24] and widely used for its flexibility in tuning different parameters such as concentration, additives, temperature, and reaction time. Furthermore, this synthesis aimed to optimize synthetic parameters in terms of yield and cost-effectiveness, and to develop a final scale-up process for possible future applications in industrial processes. In particular, the influence of two different vanadium precursors, vanadium pentoxide (V_2_O_5_) and ammonium metavanadate (NH_4_VO_3_), and two different reducing agents, citric acid monohydrate and oxalic acid, were studied. The vanadium precursor/reducing agent molar ratio was also screened, along with the reaction time and the temperature.

The synthesized samples are summarized in Table 1.

**Figure 1:**
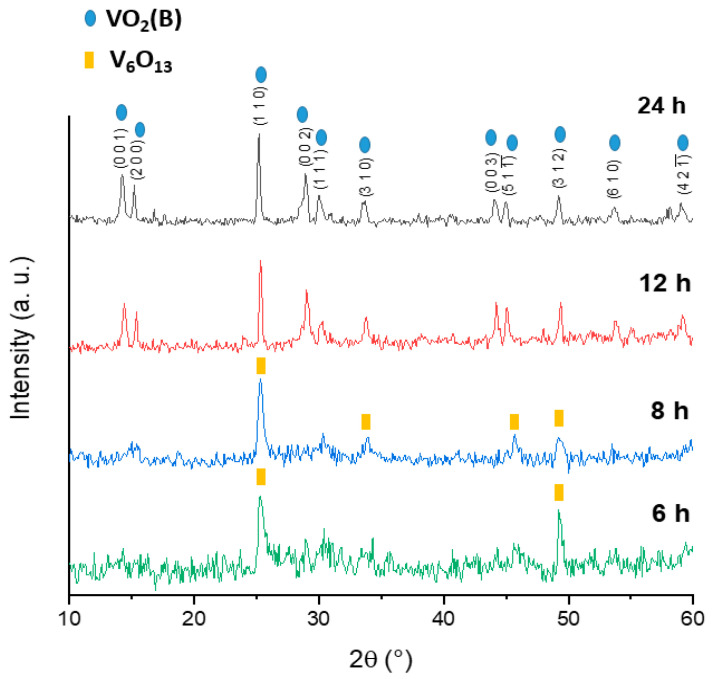
Superimposition of the XRD of the following samples: V4_1.7__24h, V5_1.7__12h, V6_1.7__8h, V7_1.7__6h (Cu anode).

First, the influence of two different vanadium precursors, i.e., V_2_O_5_ and NH_4_VO_3_, and two different reducing agents, i.e., oxalic acid and citric acid, was investigated, while the molar ratio between the vanadium precursor, the reducing agent, and the solvent was held constant. The temperature was set at 160 °C and the reaction was performed for 24 h. The V1_1.2__24h, N1_1.2__24h, V2_1.2__24h, and N2_1.2__24h samples were first investigated through XRD analysis in order to assess their crystalline phases. Figure S1 shows the X-ray diffractograms, showing an overlap of patterns deriving from a combination of different vanadium oxides (i.e., V_2_O_3_, V_4_O_7_, V_6_O_13_, V_3_O_7_). Because these samples did not fulfil the aim of this work, they were not investigated further. Subsequently, the influence of the molar ratio of vanadium precursor/reducing agent (V/R) was screened by increasing the concentration of the reducing agent from 1:1.2 to 1:1.7 [25], as shown in the sample V3_1.7__24h. This resulted in a mixed-oxide crystalline phase comparable to the V2_1.2__24h sample, as reported in Figure S2, probably ascribable to the low temperature of 160 °C. Subsequently, since the nature of both the vanadium precursor and reducing agent and the molar ratio V/R were proven to not affect the crystalline phase of the samples, the effect of the temperature was additionally investigated, based on the phase diagram of the VO_x_ system reported by Yang et al. [26]. It is known that vanadium cations V^n+^ can display multiple valence states, leading to issues related to their stabilization. While maintaining a constant molar ratio between V_2_O_5_, oxalic acid, and the solvent, the temperature was increased to 180 °C and the reaction time was systematically varied from 6 h to 24 h, in order to identify the best temperature and time required for the synthesis of pure VO_2_(B), according to the cost-effectiveness requirement. Figure 1 reports the superimposition of the XRD.

It is worth noting that 180 °C and 12 h are, respectively, the minimum temperature and minimum reaction time required for the formation of the phase-pure crystalline monoclinic phase of VO_2_(B) with space group C2/m, lattice parameters a = 12.050 Å, b = 3.693 Å, c = 6.418, α = 90°, β = 106.88°, γ = 90° (ICDD database pattern 01-081-2392). The X-ray diffraction patterns obtained at different reaction times, show that after 12 h the vanadium dioxide resulted in a highly crystalline pure phase of metastable VO_2_(B), showing relevant Bragg peaks at 14.1° (001), 15.2° (200), 28.9° (002), 30.0° (111), and 33.7° (310) that were not present in the samples after 6 h and 8 h, and a strong diffraction peak at 25.2° (110) visible in every sample. No traces of other phases or impurities were detected after 12 h and 24 h. The XRD of the sample after 24 h was comparable to the one after 12 h, whereas upon further reducing the reaction time it was possible to observe the presence of only Bragg peaks at 25.2° (110) and 49.2° (312), related to V_6_O_13_, space group C2/m. The samples treated for 6 and 8 h displayed only the reflections related to planes (110) and (312), which were in agreement with the formation of the oxide V_6_O_13_. Since the synthesis involves the reduction of V_2_O_5_ (in which the vanadium species displays an oxidation state of 5+) to VO_2_ (in which the oxidation state is 4+), V_6_O_13_ represents an intermediate crystalline phase in which vanadium atoms display an average oxidation state of 4.3. Therefore, by increasing the reaction time, vanadium is completely reduced from V^5+^ to V^4+^. The apparent mean crystallite size in the direction perpendicular to the (110) plane, resulting in D=29.0±7.3 nm and D=30.1±6.7 nm after 12 h and 24 h, respectively, shows that reaction times over 12 h did not affect the crystallite size. The Scherrer equation was employed, as reported below (1).

(1) $$\beta =\frac{K\;\cdot \;\lambda }{D\;\cdot \;\cos\theta }$$

where K = 0.94 is the shape factor, λ = 1.54 Å is the X-ray wavelength, D = crystallite size (nm), θ = Bragg angle (°).

**Figure 2:**
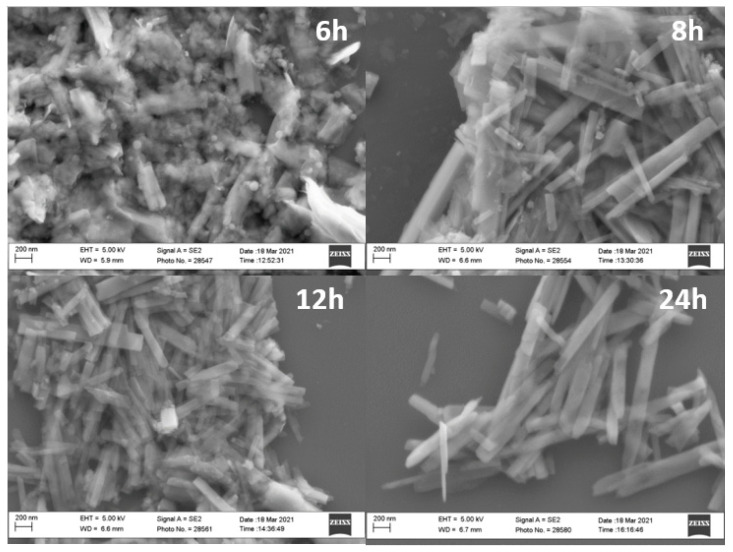
SEM measurements of the following samples: V4_1.7__6h, V5_1.7__8h, V6_1.7__12h, V7_1.7__24h, labeled as 6 h, 8 h, 12 h, and 24 h respectively.

Furthermore, it is possible to point out that upon increasing the reaction time from 6 h to 12 h, sharper (110) and (312) reflections were observed, leading to the assumption of growth of the crystallite size. In order to investigate the morphology of the samples and to evaluate whether the reaction time affected the behavior of vanadium dioxide, SEM measurements were performed on V4_1.7__24h, V5_1.7__12h, V6_1.7__8h, and V7_1.7__6h samples, as displayed in Figure 2.

It is worth noting that a modification of the morphology of vanadium dioxide was observed in relation to the systematic variation of the reaction time. For each sample, 300 nanoparticles were analyzed in order to measure the average length and width of the as-obtained VO_2_(B) rod-like structures. These structures appeared more regular after 24 h, while after 6 h, no defined shape could be observed. After 8 h and 12 h, the particles had the same average length and width, i.e., 651 ± 104 nm and 401 ± 38 nm, respectively, whereas after 24 h, the rod-like structures appeared longer and more regular, with an average length and width of 1136 ± 127 nm and 407 ± 41 nm, respectively. While the widths of the V5_1.7__8h, V6_1.7__12h, and V7_1.7__24h samples did not increase upon increasing the reaction time, the length doubled. Therefore, since the aim of the synthesis was to obtain a pure VO_2_(B) crystalline phase without any specific morphology requirement, the V6_1.7__12h sample was selected for the following thermal conversion to the VO_2_(M1) polymorph.

### 2.2. Conversion of VO_2_(B) to VO_2_(M1) Polymorph

**Figure 3:**
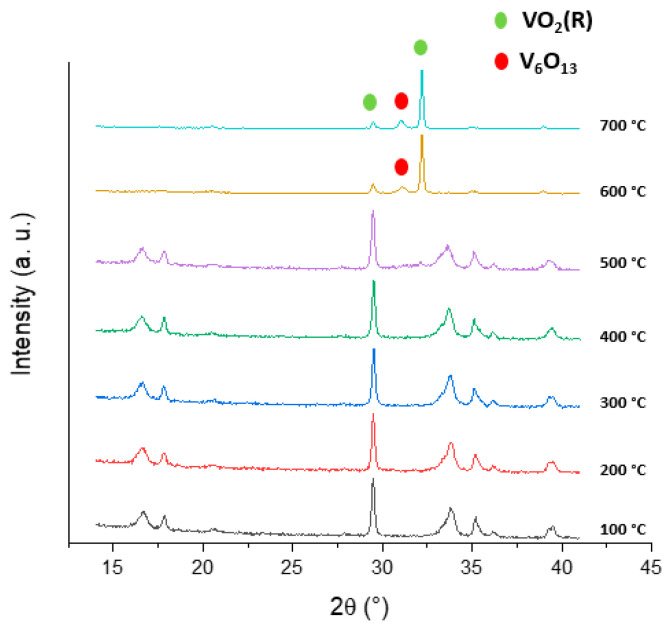
Superimposition of the temperature-dependent XRD patterns of the V5_1.7__12h sample (Co anode).

The VO_2_(B) polymorph structure consists of VO_6_ octahedral units joined by edges to form strings, which are connected by corners to give a three-dimensional structure. VO_2_(M1) displays the same symmetry as the VO_2_(B) polymorph except for the orientations of the oxygen octahedra, in which their 4-fold axes are aligned along two perpendicular planes [27]. In order to identify the optimal temperature required for the whole conversion of the VO_2_(B) polymorph into the VO_2_(M1) structure, a temperature-dependent XRD analysis was performed on the V6_1.7__12h sample. Figure 3 shows the superimposition of the X-ray patterns from 25 °C to 700 °C. Since the metal–insulator transition occurred at 68 °C [14], the VO_2_(M1) polymorph existed at low temperature, while the VO_2_(R) crystal structure was present at temperatures higher than 68 °C, as can be seen from the XRD at 600 °C and 700 °C.

It is worth noting that up to 500 °C, the XRD displayed the reflections of pure VO_2_(B), while at 600 °C, the pattern matched well with the VO_2_(R) phase, with a small amount of the oxidized phase V_6_O_13_. Building on the data obtained from the temperature-dependent XRD analyses, the sample V6_1.7__12h was annealed in a furnace in order to induce the formation of the VO_2_(M1) crystalline phase by varying the temperature and the reaction time, as reported in Table 2. The temperature was increased from 550 °C up to 700 °C and the time was varied from 2 h to 4.5 h under nitrogen to avoid the formation of the V_6_O_13_ [28] phase.

**Figure 4:**
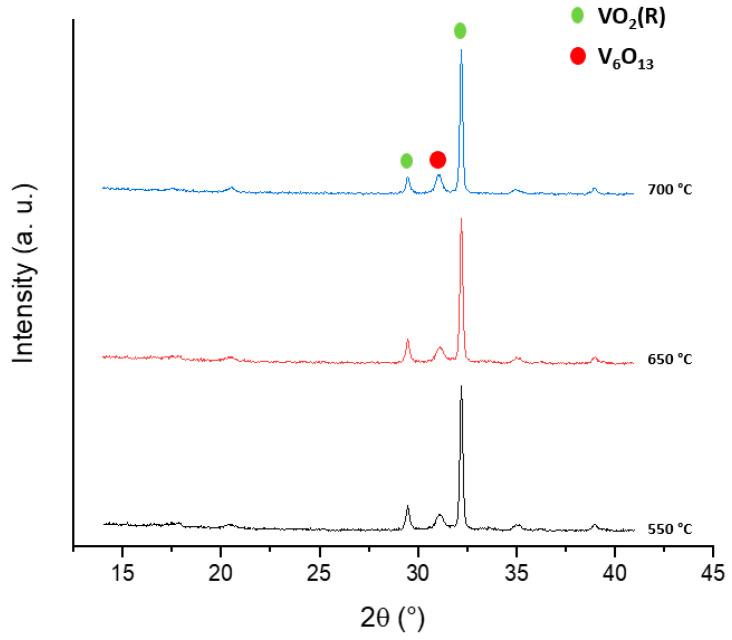
Superimposition of the XRD patterns of V5_1.7__12h sample annealed at 550 °C, 650 °C, and 700 °C (Co anode).

Figure 4 shows the superimposition of the patterns of V5_1.7__12h treated at 550 °C and at 700 °C.

**Figure 5:**
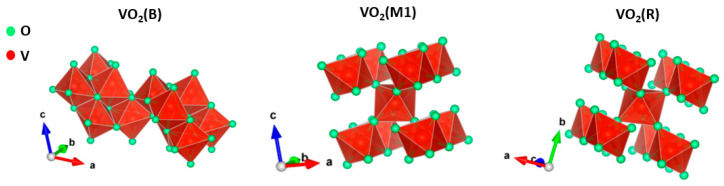
Comparison between the structures of VO_2_(B), VO_2_(M1), and VO_2_(R).

The mechanism involved in the conversion of VO_2_(M1) to VO_2_(R) has always been a very controversial topic, since the lattice distortion that causes the MIT has not yet been clarified [21]. Two mechanisms have been proposed, the Peierls and Mott–Hubbard models. The first is linked to an electron–lattice interaction [26] that arises from a lattice structural change in the material that causes a band structure change, and the second is related to an electron–electron interaction [29] that is often induced by temperature. Vanadium dioxide exhibits the possible formation of three different polymorphs during the reversible conversion from VO_2_(M) to VO_2_(R), i.e., VO_2_(M1), VO_2_(T), and VO_2_(M2), as a consequence of the potential presence of strain within the crystal structure [22]. In particular, in the M1 phase, the V^4+^ atoms are paired and tilted, forming zigzag chains along the c_R_ axis. In contrast, the VO_2_(M2) polymorph has two distinct sublattices of vanadium atoms: in sublattice A, the V^4+^ atoms are paired but not tilted along the c_R_ axis, and in sublattice B they are tilted perpendicular to the c_R_ axis but unpaired [22]. Aside from that, VO_2_(M1) was the most prevalent polymorph obtained during the transition. It has a distorted rutile structure, in which the band structure comprises V_3d_ and O_2p_ orbitals. O_2p_ orbitals are located at 2.5 eV below the Fermi level and include σ and π bonds, while vanadium cations V^4+^ provide 3d orbitals that are split into triply degenerate $t_{2g}$ states, i.e., $d_{xz}$, $d_{xy}$, $d_{yz}$, and doubly degenerate $e_{g}$ states, i.e., $d_{x^{2}-y^{2}}$, $d_{z^{2}}$, which are placed at higher energy compared to the $t_{2g}$ orbitals. The crystal field theory predicts the splitting of $t_{2g}$ orbitals into $d_{\left|\right|}$ and $\pi^{*}$; due to the V–V twisting, in the monoclinic M1 phase, the $d_{\left|\right|}$ band is split into two energy bands, i.e., $d_{\left|\right|}$ and $d_{\left|\right|}^{*}$, and a forbidden band is formed between them of nearly 0.7 eV. The Fermi level lies between the $d_{\left|\right|}$ and the $\pi^{*}$ orbitals causing insulator behavior. In contrast, in the rutile phase, the Fermi level of the VO_2_(R) polymorph lies between the $d_{\left|\right|}$ and the $\pi^{*}$ bands, showing metallic behavior [22]. During the transition from to VO_2_(M1) to VO_2_(R), a charge in the crystal lattice occurs, inducing the switch from a low-symmetry monoclinic structure to a high-symmetry tetragonal structure. In the VO_2_(R) polymorph, the distance between two nearest V–V is 2.85 Å, while in the VO_2_(M1) crystal structure V–V bonds with different lengths are formed. The longest distance is 3.19 Å and the shortest is 2.60 Å. Figure 5 shows the comparison between the structures of VO_2_(B), VO_2_(M1), and VO_2_(R) polymorphs.

**Figure 6:**
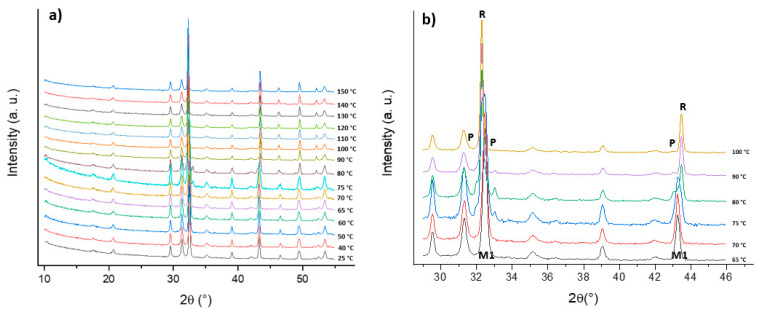
Overlap of the temperature-dependent XRD analysis from 25 °C to 150 °C (**a**), and a focus from 65 °C to 100 °C and from to 29° (2θ) to 46° (2θ) (**b**). The crystalline phases are labeled in (**b**) as M1, R, and P for VO_2_(M1), VO_2_(R), and VO_2_(P), respectively (Co anode).

Since the MIT is reported in the literature to occur at 68 °C, a temperature-dependent XRD analysis was carried out in the range of 25 °C to 150 °C to identify the temperature of the transition from the obtained VO_2_(M1) to VO_2_(R), as reported in Figure 6.

**Figure 7:**
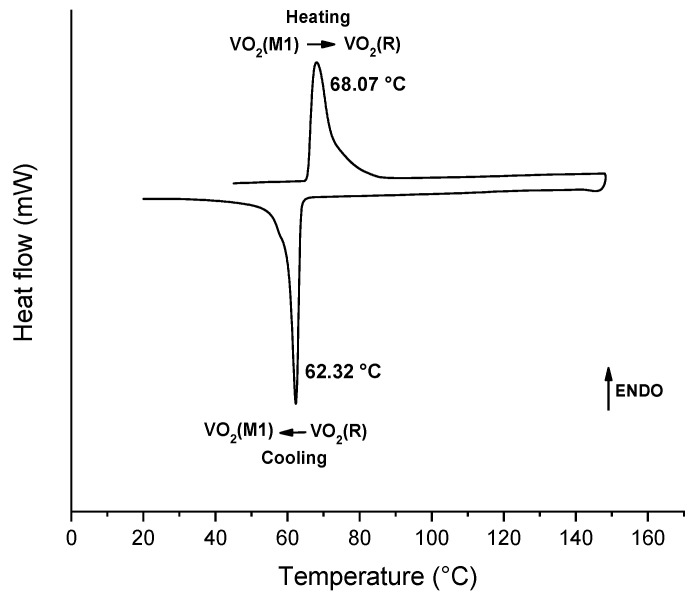
DSC curve of V6_1.7__12h sample over two heating and cooling cycles, where 68.07 °C and 62.32 °C were the temperatures of the phase transitions VO_2_(M) → VO_2_(R) and VO_2_(M) → VO_2_(R), respectively.

The metal–insulator transition was revealed by a shift of two Bragg reflections belonging to VO_2_(M1): the first at 32.5° underwent a shift to a lower angle (from 32.5° to 32.3°), while the reflection at 43.3° underwent a shift to a higher angle (from 43.4° to 43.5°). During the MIT, the formation of an orthorhombic phase, an additional polymorph known as VO_2_(P) [20] and labeled as P in Figure 6b, was observed. This species appeared during the conversion from VO_2_(M1) to VO_2_(R) and disappeared when the transition was completed. Therefore, the thermal conversion of the monoclinic vanadium dioxide to the tetragonal crystal structure occurred over a broad range between 65 °C and 100 °C. Since the reversible conversion from VO_2_(M1) to VO_2_(R) is a fundamental characteristic of vanadium dioxide, DSC analysis was carried out in order to identify the actual temperature of the phase transition (Figure 7).

As can be seen, the two phase transitions (M1) → (R) and (R) → (M1) occurred at 68.1 °C and 62.3 °C, respectively, and the transition enthalpies (ΔH_T_) associated with the conversions were 2.8 kJ/mol and −2.7 kJ/mol during the heating and cooling cycles, respectively. In particular, the phase transition (M1) → (R) consists of an endothermic phenomenon, while the (R) → (M1) transition involves an exothermic phenomenon, according to the literature [30]. The peaks show some broadening and a shoulder is apparent, specifically at about 55 °C in the cooling exotherm. This can probably be ascribed to the polydispersity in the size of crystals, supported by the SEM images in Figure 2.

### 2.3. Functionalization of VO_2_(M1) with a Silica Layer

**Figure 8:**
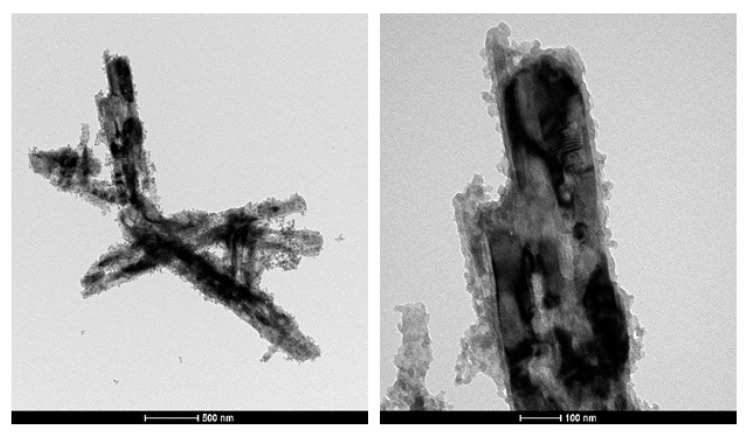
TEM images of V5_1.7__12h sample (the black rod-like structures) functionalized with a SiO_2_ layer (the grey globular layer).

Since the VO_2_(M1) could be applied for possible use in dynamic concrete, a functionalization with TEOS to produce a silica coating was carried out, with the purpose of increasing the compatibility between the inorganic powder and the concrete itself. This coating was optimized for the V5_1.7__12h sample after annealing treatment at 700 °C. The literature regarding this functionalization procedure is not extensive. Some works, reported by Zhu et al. [31] and Zhe at al. [32], describe a functionalization route aimed at coating VO_2_ nanoparticles with silica using TEOS as a precursor. In our work, TEM analysis was employed in order to detect the presence of the SiO_2_ layer, as reported in Figure 8.

**Figure 9:**
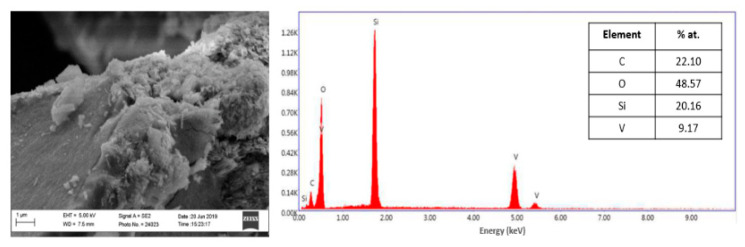
SEM-EDX analysis on V5_1.7__12h sample functionalized with silica.

The amorphous and globular layer around the rod-like crystals of vanadium dioxide was ascribed to the presence of the SiO_2_, with a thickness up to 30 nm. The coating seemed to be homogeneously distributed on the surface of VO_2_ crystals. In order to verify the homogeneity of the functionalization around the vanadium dioxide particles, a SEM–EDX analysis was carried out, as reported in Figure 9.

The energy dispersive X-ray (EDX) analysis provided additional insight into the composition of the surface of vanadium dioxide coated with the silica layer. The results confirmed the presence of vanadium (V), oxygen (O), and silicon (Si) elements, in particular with an atomic ratio Si/V of 2.2, in agreement with the ratio employed in the synthesis. Subsequently, since the pH of concrete is around pH = 11, after the incorporation of clinker, the stability of the functionalized V5_1.7__12h sample under alkaline pH was evaluated by suspending 100 mg of powder in a Ca(OH)_2_ solution under vigorous stirring. After 24 h, an XRD analysis was carried out, which did not show the formation of any additional crystalline phases, as reported in Figure S3; therefore, the functionalized vanadium dioxide can be used for possible embedding into a concrete matrix. Additionally, a scale-up process was implemented in order to increase the amount of VO_2_(B) obtained from the hydrothermal synthesis. With this purpose, the concentration of the vanadium precursor and the reducing agent was increased while the V_2_O_5_/oxalic acid molar ratio was kept constant. XRD measurements were employed in order to exclude the formation of other crystalline phases beyond the VO_2_(B), and these confirmed the phase purity of monoclinic vanadium dioxide.

## 3. Materials and Methods

Vanadium (V) pentoxide (V_2_O_5_, 98%) and ammonium metavanadate (V) (NH_4_VO_3_, 99.9%) were employed as precursors for the synthesis of vanadium dioxide, while oxalic acid (98%) and citric acid monohydrate (≥99%) were used as reducing agents. Deionized water was employed as the solvent. Ammonium hydroxide solution (33% h), tetraethyl orthosilicate (TEOS), and absolute ethanol (99%) were employed for the functionalization procedure, while Ca(OH)_2_ was employed for the stability test in alkaline pH. All reagents were purchased from Sigma-Aldrich.

### 3.1. Hydrothermal Synthesis and Scale-Up Process

A given amount of vanadium precursor (V_2_O_5_ or NH_4_VO_3_) and of reducing agent (oxalic acid or citric acid) were added to 10 mL of deionized water in a molar ratio of 1:1.2 and 1:1.7 vanadium precursor/reducing agent, and placed in a 23 mL A255AC PTFE cup with constant stirring. The solution was stirred for 20 min and then placed in a stainless steel 4745 General Purpose Acid-Digestion Bomb and heated at a set temperature, from 160 °C to 180 °C, for a set time, from 6 h to 24 h, in order to reduce the V_2_O_5_ (V^5+^) to VO_2_ (V^4+^), as reported in Equation (2) below:

(2) $${\text{V}}_{2}{\text{O}}_{5}+{\text{H}}_{2}{\text{C}}_{2}{\text{O}}_{4}\to 2{\text{VO}}_{2}+2{\text{CO}}_{2}+{\text{H}}_{2}\text{O}$$

After cooling at room temperature, the resulting powder was washed three times with 5 mL of deionized water and once with 5 mL of ethanol and collected by centrifugation at 10,000 rpm for 10 min before drying at 80 °C in an oven for 6 h. The synthesis was scaled up by increasing the concentrations of the precursors in the solution, therefore constantly decreasing the molar ratio with the solvent, and by using a 210 mL PTFE cup placed in a stainless steel Acid-Digestion Bomb. The prepared samples are summarized in the Results and Discussion section, described in detail in the supporting information in Table S1 and labeled in the paper as follows:

Vx_y__z, where V = V_2_O_5_ precursor, x = number of the synthetized sample, y = V_2_O_5_: reducing agent molar ratio, z = reaction time
Nx_y__z, where N = NH_4_VO_3_ precursor, x = number of the synthetized sample, y = NH_4_VO_3_: reducing agent molar ratio, z = reaction time

### 3.2. Thermal Conversion of VO_2_(B) to VO_2_(M1)

The thermal treatment, that aimed at the conversion of VO_2_(B) into VO_2_(M1), was carried out via annealing under pure nitrogen atmosphere in a tube furnace at a set temperature, from 550 °C to 700 °C, for a set time, from 2 h to 4.5 h, with a ramp of 5 °C/min. At the end, the powder was recovered and stored for further analysis.

### 3.3. Functionalization of VO_2_(M1) with SiO_2_ Layer

A given amount of VO_2_(M1) powder was dispersed in an aqueous solution of 5 M citric acid in a molar ratio of 1:1 and 1:4 VO_2_(M)/citric acid, and the pH value was then adjusted to pH 5 with the addition of ammonium hydroxide solution (33% *w*/*w*) under constant stirring for 2 h. The pH was then increased to pH 10–11 by adding NH_3_, and a solution of 0.045 M tetraethyl orthosilicate (TEOS) in ethanol was added to the suspension. The mixture was stirred for a set time, from 16 h to 24 h, to complete the hydrolysis and condensation of TEOS. The suspension was filtered, and the obtained powder was washed several times with deionized water to neutral pH and dried in the oven at 60 °C for 8 h.

### 3.4. Stability Tests at Alkaline pH

A given amount of VO_2_(M1) functionalized with SiO_2_ was suspended in 30 mL of aqueous solution of 2 mM Ca(OH)_2_ at pH = 11 for 24 h under vigorous stirring at room temperature. The sample was allowed to dry in the oven at 90 °C for 6 h.

### 3.5. Characterizations of VO_2_(B)

#### 3.5.1. X-ray Diffraction Analysis

XRD patterns were collected with a Bruker D8 Advance diffractometer equipped with a Göbel mirror and employing a Cu K_α_ radiation source. All patterns were recorded in the 10–60° range with a 0.01° (2θ) scan step and a 10 s per step acquisition time. The temperature dependent-XRD patterns were collected with a Panalytical X’Pert Pro diffractometer, equipped with a high-temperature chamber (ANTON PAAR HTK 16) and employing a Co K_α_ radiation source. All patterns were recorded in the 14–41° range with a 0.03° (2θ) scan step and a 60 s per step acquisition time. Patterns were analyzed using HighScore Plus and EVA software in order to obtain the lattice parameters and the apparent mean crystallite sizes of the powders.

#### 3.5.2. SEM-EDX Analysis

Field-emission scanning electron microscopy (FE-SEM) and energy-dispersive X-ray analysis (EDX) were run on a Zeiss SUPRA 40VP equipped with an Oxford INCA x-sight X-ray detector. Morphological analysis was carried out by setting the acceleration voltage at 5 kV, whereas the EDX compositional investigations were obtained by setting the acceleration voltage at 20 kV. Particle size analysis was carried out using the public domain software ImageJ (National Institute of Health, Bethesda, MD, USA). A total of 300 particles were analyzed to extract the width and length of the particles.

#### 3.5.3. TEM Analysis

TEM micrographs were obtained with a FEI Tecnai G12 microscope operating at 100 kV, equipped with an OSIS Veleta camera. Samples were prepared by suspending the dried powders in MilliQ water through sonication and then depositing them on 300-mesh lacey carbon-coated copper grids.

#### 3.5.4. DSC Analysis

All measurements were carried out with a model 2920 calorimeter (TA Instruments) operating in air. The samples, weighing about 5–7 mg, were closed in aluminum pans. A first heating ramp at 10 °C/min was used from 25 °C up to 150 °C and, after erasure of thermal history by a 5 min isotherm at 150 °C, the sample was cooled down to 25 °C at 10 °C/min and heated again to 150 °C at the same rate. High-purity indium was used to calibrate the DSC temperature and enthalpy scales.

## 4. Conclusions

In this study, the synthesis of pure vanadium dioxide VO_2_(B), its thermal conversion to VO_2_(M1), and its further functionalization with a silica layer are reported. In particular, a pure crystalline phase of monoclinic VO_2_(B) was produced via a straightforward, reproducible, and scalable subcritical hydrothermal route, followed by an annealing process under nitrogen to induce the formation of the VO_2_(M1) polymorph.

Compared to the syntheses reported in literature, our approach is easy, fast, cost-effective, and uses no hazardous chemicals.

Using a combination of different analytical tools and based on XRD analyses, SEM, TEM, and EDX measurements for structural, morphological, and compositional characterization respectively, it was possible to confirm the purity of the crystalline phase, the size of the rod-like particles, and the presence of the silica layer. By varying the synthetic parameters in terms of vanadium precursor/reducing agent molar ratio, temperature, and reaction time, it was established that 12 h and 180 °C are the optimal time and temperature required for the formation of a pure crystalline phase of VO_2_(B), according to the requirements of high yield and a cost-effective process. The thermal conversion and subsequent functionalization with a silica layer were crucial to obtaining the desired thermochromic phase VO_2_(M1) with enhanced stability in an alkaline environment, disclosing the possibility of its use in a concrete matrix for possible dynamic cooling effect. Furthermore, it is worth noting that the scale-up process, fundamental for future application in industrial processes, did not affect either the morphology or the purity of the crystalline phase, or the high stability of the vanadium dioxide in an alkaline environment.

## Figures and Tables

**Table 1:** List of the samples synthesized via hydrothermal synthesis by changing the vanadium precursors, the molar ratios vanadium precursor (V)/reducing agent (R)/solvent (S), the temperature, and the reaction time.

Samples	Vanadium Precursor (V)	Reducing Agent (R)	Molar Ratio (V:R:S)	Temperature (°C)	Time (h)	XRD Output
V1_1.2__24h	V_2_O_5_	Citric acid monohydrate	1:1.2:605	160	24	Mixed oxides
N1_1.2__24h	NH_4_VO_3_					Mixed oxides
V2_1.2__24h	V_2_O_5_	Oxalic acid				Mixed oxides
N2_1.2__24h	NH_4_VO_3_					Mixed oxides
V3_1.7__24h	V_2_O_5_	Oxalic acid	1:1.7:605	160	24	Mixed oxides
V4_1.7__6h	V_2_O_5_	Oxalic acid	1:1.7:605	180	6	V_6_O_13_
V5_1.7__8h					8	V_6_O_13_
V6_1.7__12h					12	VO_2_(B)
V7_1.7__24h					24	VO_2_(B)
V8_1.7__12h	V_2_O_5_	Oxalic acid	1:1.7:605	180	12	VO_2_(B)
V9_1.7__12h			1:1.7:509			
V10_1.7__12h			1:1.7:424			
V11_1.7__12h			1:1.7:364			
V12_1.7__12h			1:1.7:318			
V13_1.7__12h			1:1.7:282			

**Table 2:** Annealing at different temperatures and times for the conversion of VO_2_(B) to VO_2_(M1).

Sample	Temperature (°C)	Time (h)
V5_1.7__12h	550	2
	550	4.5
	650	4.5
	700	4.5
	700	2

## Data Availability

The data presented in this study are available within the paper and the Supplementary Information.

## Supplementary Materials

The following are available online, Table S1: Detailed list of the synthetized samples through hydrothermal route, Figure S1 XRD analyses of the following samples: V1_1.2__24h, N1_1.2__24h, V2_1.2__24h, N2_1.2__24h, Figure S2: XRD analyses of V2_1.2__24h (on the left) and V3_1.7__24h (on the right) with two different vanadium precursor/reducing agent molar ratios, e.g., 1:1.2 and 1:1.7 respectively, without showing any improving in the formation of the pure crystalline phase, Figure S3: XRD analyses before (black line) and after (red line) the stability test of vanadium dioxide by its suspension in an alkaline solution of Ca(OH)_2_ for 24 h.

## Sample Availability

All samples are available from the authors.
