# Supplementary material for: Systematic Exploration of the Synthetic Parameters for the Production of Dynamic VO2(M1)
Source: Molecules. 2021 Jul 27;26(15):4513. doi: 10.3390/molecules26154513 (PMC8348029; doi:10.3390/molecules26154513)
Supplement: Supplementary file 1 [file molecules-26-04513-s001.zip › molecules-1250535-supplementary.pdf]

Supporting Information

Giulia Bragaggia[1,2], Andrea Cacciatore[1,3], Elisa Poffe[1,2], Claudia Capone[3], Federico Zorzi[4,5], Valerio Causin[1], Silvia Gross[*1,2]

[1] Dipartimento di Scienze Chimiche, Università degli Studi di Padova, via Marzolo 1, 35131 Padova, Italy

[2] INSTM, Consorzio Interuniversitario per la Scienza e Tecnologia dei Materiali, via Giusti 9, 50121, Firenze, Italy

[3] Italcementi S.p.A., HeidelbergCement Group, via Stezzano 87, 24126, Bergamo, Italy

[4] CEASC, Centro di Analisi e Servizi per la Certificazione, via Jappelli 1/A 35131 Padova, Italy

[5] Dipartimento di Geoscienze, Università degli Studi di Padova, via Gradenigo 6, 35131 Padova, Italy

* Correspondence: silvia.gross@unipd.it

Hydrothermal synthesis

Table 1: : Detailed list of the synthetized samples through hydrothermal route.

Samples	Reagents	Quantities	PM (g/mol)	Density (g/cm³)	moles	Concentration (M=mol/mL)	Molar ratios	Temperature (°C)	Time (h)
**V1(1.2)_24h**	V_2O_5	0.1670 g	181.88		0.000918	0.0000918	1	160	24
	Citric acid	0.2376 g	210.14		0.001131	0.0001131	1.2		
	Deionized H_2O	10 mL	18.00	1.000	0.555556		605		
**N1(1.2)_24h**	NH_4VO_3	0.1666 g	181.88		0.000916	0.0000916	1	160	24
	Citric acid	0.2368 g	210.14		0.001127	0.0001127	1.2		
	Deionized H_2O	10 mL	18.00	1.000	0.555556		607		
**V2(1.2)_24h**	V_2O_5	0.1673 g	181.88		0.000920	0.0000920	1	160	24
	Oxalic Acid	0.0987 g	90,034		0.001096	0.0001096	1.2		
	Deionized H_2O	10 mL	18.00	1.000	0.555556		604		
**N2(1.2)_24h**	NH_4VO_3	0.1064 g	181.88		0.000585	0.0000585	1	160	24
	Oxalic Acid	0.0635 g	90,034		0.000705	0.0000705	1.2		
	Deionized H_2O	10 mL	18.00	1.000	0.555556		950		
**V3(1.7)_24h**	V_2O_5	0.1679 g	181.88		0.000923	0.0000923	1	160	24
	Oxalic Acid	0.1420 g	90,034		0.001577	0.0001577	1.7		
	Deionized H_2O	10 mL	18.00	1.000	0.555556		602		
**V4(1.7)_24h**	V_2O_5	0.1687 g	181.88		0.000928	0.0000928	1	180	24
	Oxalic Acid	0.1425 g	90,034		0.001583	0.0001583	1.7		
	Deionized H_2O	10 mL	18.00	1.000	0.555556		599		
**V5(1.7)_12h**	V_2O_5	0.1672 g	181.88		0.000919	0.0000919	1	180	12
	Oxalic Acid	0.1416 g	90,034		0.001573	0.0001573	1.7		
	Deionized H_2O	10 mL	18.00	1.000	0.555556		604		

**V6(1.7)_8h**	V_2O_5	0.1686 g	181.88		0.000927	0.0000927	1	180	8
	Oxalic Acid	0.1427 g	90,034		0.001585	0.0001585	1.7		
	Deionized H_2O	10 mL	18.00	1.000	0.555556		599		
**V7(1.7)_6h**	V_2O_5	0.1677 g	181.88		0.000922	0.0000922	1	180	6
	Oxalic Acid	0.1418 g	90,034		0.001575	0.0001575	1.7		
	Deionized H_2O	10 mL	18.00	1.000	0.555556		603		

XRD analyses

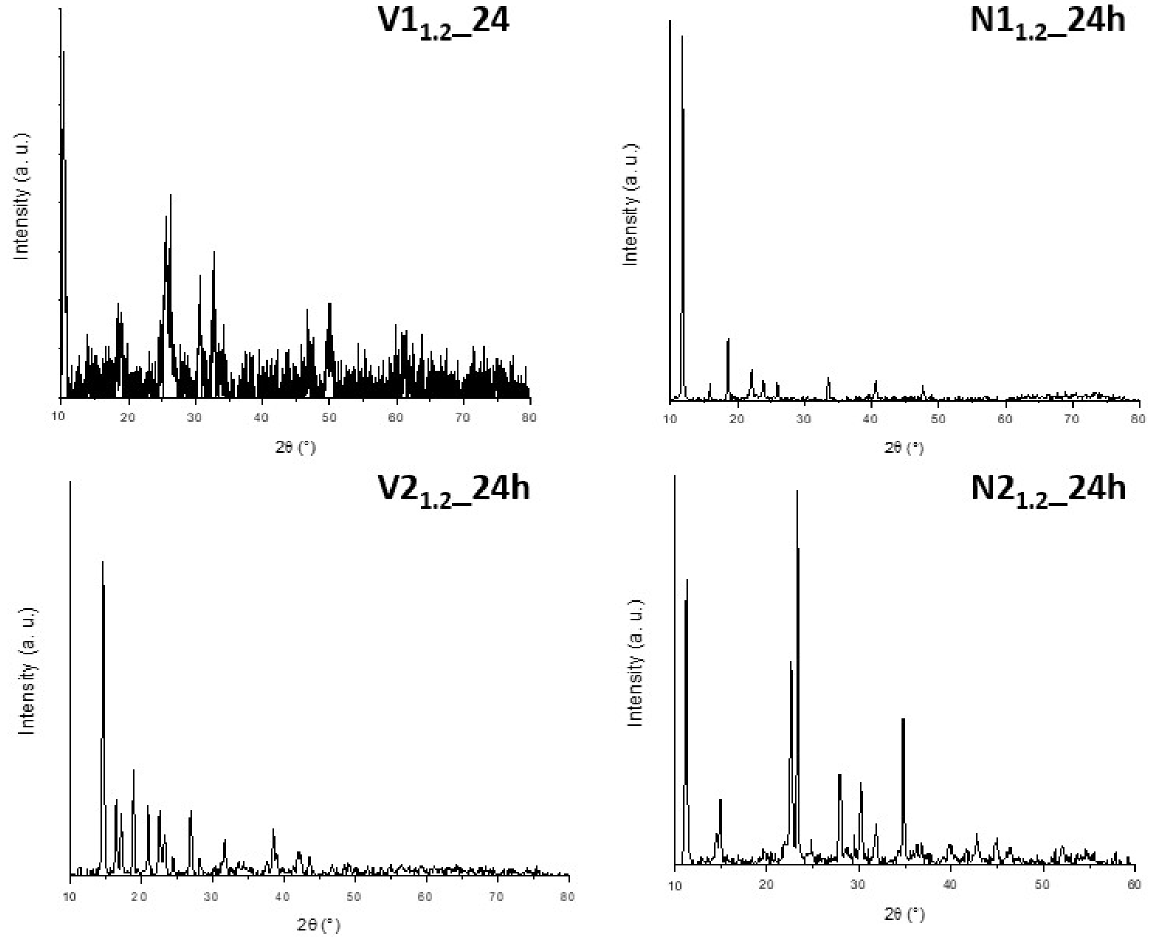

Figure S1: XRD analyses of the following samples: V1$_{1.2}$_24h, N1$_{1.2}$_24h, V2$_{1.2}$_24h, N2$_{1.2}$_24h.

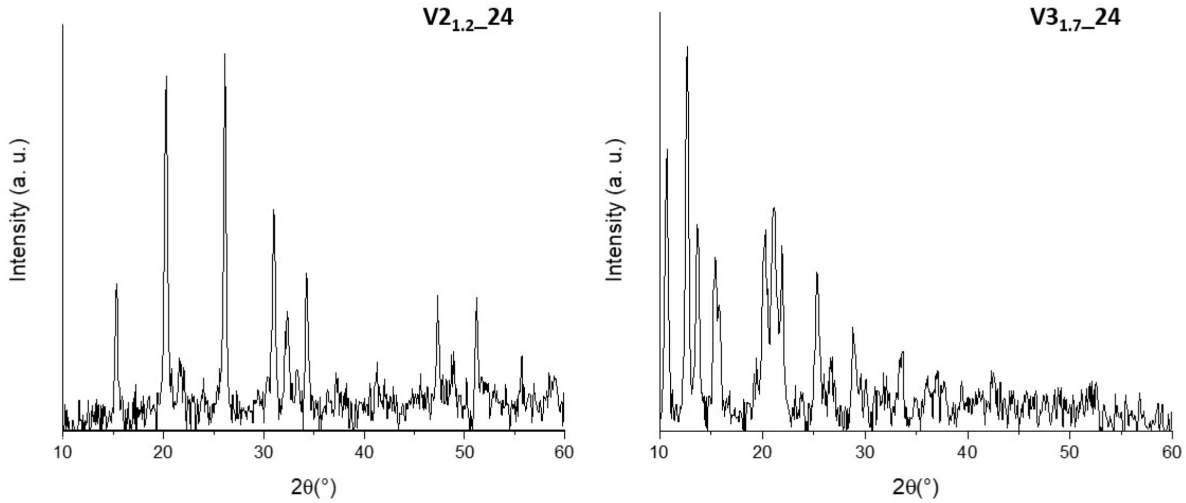

Figure S2: XRD analyses of V2$_{1.2}$_24h (on the left) and V3$_{1.7}$_24h (on the right) with two different vanadium precursor/reducing agent molar ratios, e.g. 1:1.2 and 1:1.7 respectively, without showing any improvement in the formation of the pure crystalline phase VO_2(B).

Stability test in alkaline pH

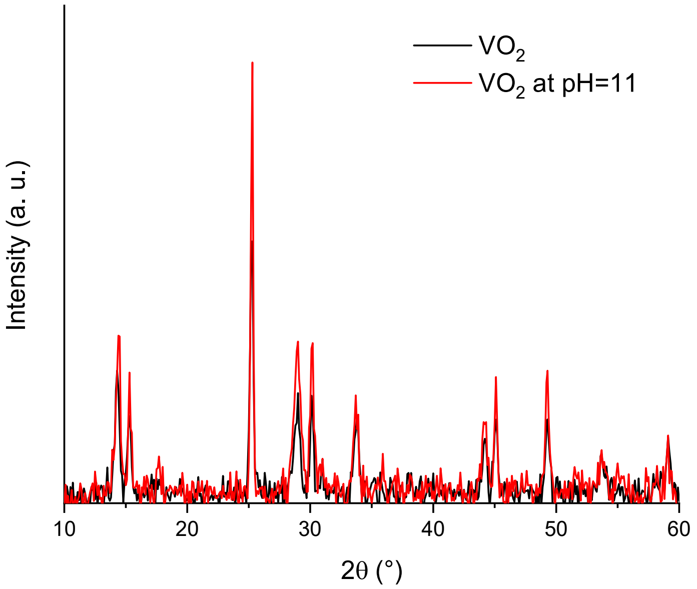

Figure S3: XRD analyses before (black line) and after (red line) the stability test of vanadium dioxide, by its suspension in an alkaline solution of $Ca(OH)_2$ at pH = 11 for 24 hours.

SEM measurements

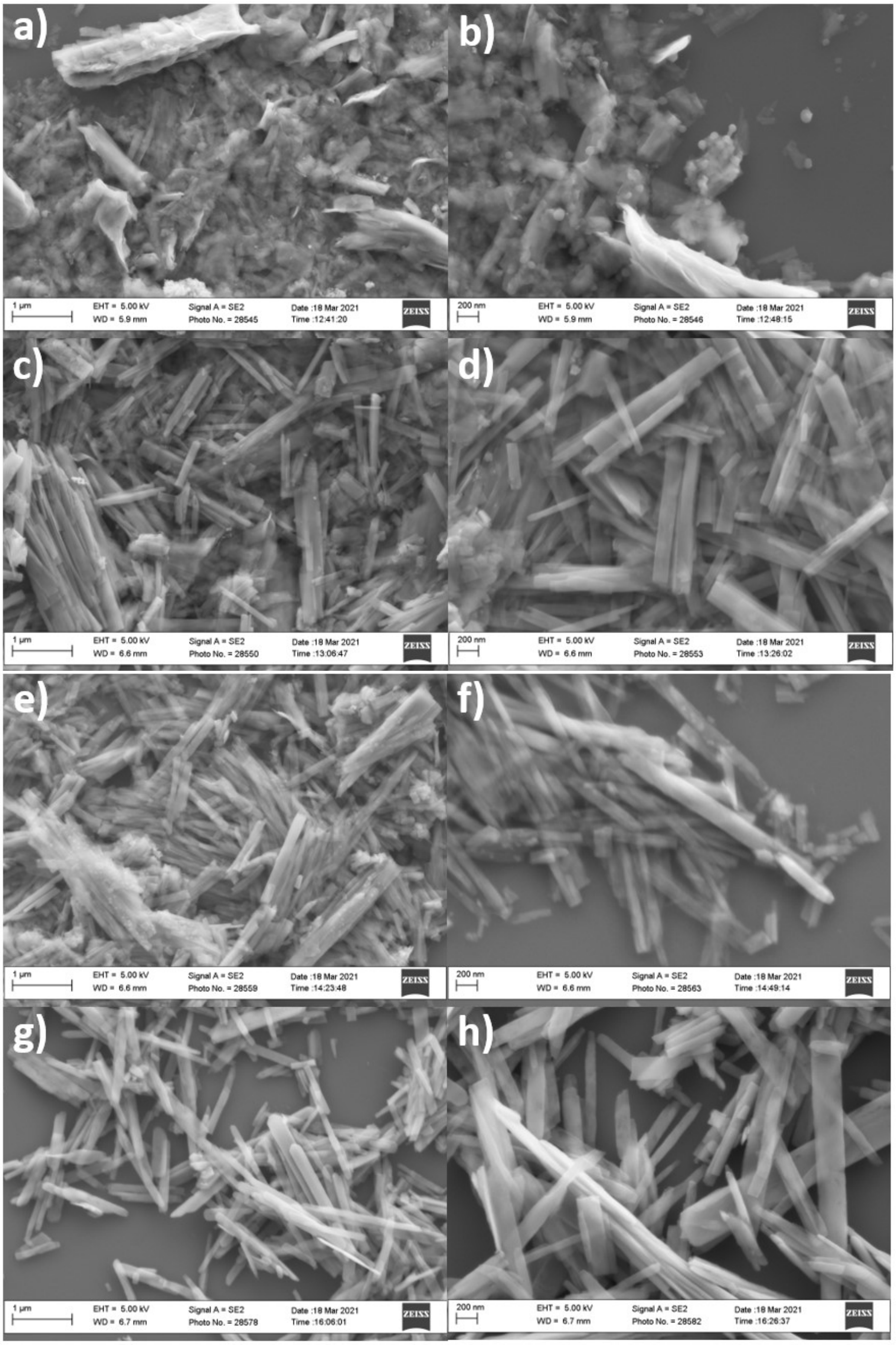

Figure S4: SEM images of the synthetized samples after a reaction time of 6 hours, a) and b), 8 hours, c) and d), 12 hours, e) and f), and 24 hours, g) and h).
